# Endemic *Juniperus* Montane Species Facing Extinction Risk under Climate Change in Southwest China: Integrative Approach for Conservation Assessment and Prioritization

**DOI:** 10.3390/biology10010063

**Published:** 2021-01-17

**Authors:** Mohammed A. Dakhil, Marwa Waseem A. Halmy, Walaa A. Hassan, Ali El-Keblawy, Kaiwen Pan, Mohamed Abdelaal

**Affiliations:** 1 Botany and Microbiology Department, Faculty of Science, Helwan University, Cairo 11790, Egypt; 2 CAS Key Laboratory of Mountain Ecological Restoration and Bioresource Utilization & Ecological Restoration Biodiversity Conservation Key Laboratory of Sichuan Province, Chengdu Institute of Biology, Chinese Academy of Sciences, Chengdu 610041, China; pankw@cib.ac.cn; 3 University of Chinese Academy of Sciences, Beijing 100039, China; 4 Department of Environmental Sciences, Faculty of Science, Alexandria University, Alexandria 21511, Egypt; 5 Department of Biology, College of Science, Princess Nourah bint Abdulrahman University, Riyadh P. O. Box 84428, Saudi Arabia; wahassan@pnu.edu.sa; 6 Department of Applied Biology, Faculty of Science, University of Sharjah, Sharjah P. O. Box 27272, UAE; akeblawy@sharjah.ac.ae; 7 Department of Botany, Faculty of Science, Mansoura University, Mansoura 35516, Egypt; mohamed_eco@mans.edu.eg

**Keywords:** ensemble modelling, AOO, IUCN red list, alpine endemic species, global warming, biodiversity hotspots

## Abstract

**Simple Summary:**

Climate change is one of the most significant drivers of habitat loss and species extinction, particularly montane endemic species such as Juniper trees, which are restricted to unique habitats. Therefore, assessing the impact of climate change on the extinction risk of species is a promising tool or guide for species conservation planning. The loss in species habitat due to global warming indicates the level of extinction or endangerment. Predictions of suitable habitats are outputs from assessment analysis. This will help conservationists discover new populations of endemic species and help raise the awareness of local people to save and rescue these endangered species.

**Abstract:**

Climate change is an important driver of biodiversity loss and extinction of endemic montane species. In China, three endemic *Juniperus* spp. (*Juniperus* *pingii* var. pingii, *J.* *tibetica*, and *J.* *komarovii*) are threatened and subjected to the risk of extinction. This study aimed to predict the potential distribution of these three *Juniperus* species under climate change and dispersal scenarios, to identify critical drivers explaining their potential distributions, to assess the extinction risk by estimating the loss percentage in their area of occupancy (AOO), and to identify priority areas for their conservation in China. We used ensemble modeling to evaluate the impact of climate change and project AOO. Our results revealed that the projected AOOs followed a similar trend in the three *Juniperus* species, which predicted an entire loss of their suitable habitats under both climate and dispersal scenarios. Temperature annual range and isothermality were the most critical key variables explaining the potential distribution of these three *Juniperus* species; they contribute by 16–56.1% and 20.4–38.3%, respectively. Accounting for the use of different thresholds provides a balanced approach for species distribution models’ applications in conservation assessment when the goal is to assess potential climatic suitability in new geographical areas. Therefore, south Sichuan and north Yunnan could be considered important priority conservation areas for in situ conservation and search for unknown populations of these three *Juniperus* species.

## 1. Introduction

In the last one hundred years, the average global temperature has increased by approximately 0.74 °C, and the warming trend is projected to increase in the coming decades to reach 2.8–5.3 °C by 2085 [1]. The distribution of plants is affected by global warming and tends to move towards suitable habitats, whether northward or upward in elevation [2]. Climate change may lead to contractions in population sizes that could lead to local species extinction [3]. Moreover, drought, which is one outcome of global climate change, will affect the habitat suitability of species [4].

In this context, plants adapted to cold conditions at the top of mountains, particularly endemics, are at the highest risk of extinction [5]. Endemic plants are restricted to a specific range in well-defined areas [6]. Therefore, their conservation is necessary at the global, national, and local scales [7]. The decline in species population sizes and suitable habitats enhances extinction risk, particularly for narrow-range endemic species [8]. Engler et al. [5] predicted that nearly 30–50% of the species distributed at high altitudes would lose 80% of their suitable habitats by 2070–2100 [5].

There are 244 species of conifer in China, belonging to 23 genera and four families (Pinaceae, Cupressaceae, Taxaceae, and Podocarpaceae), out of which eight genera with 115 species are endemic. Cupressaceae and Pinaceae are the largest two families with endemic species in China [9]; they consist of 21.73% and 63.47% of all endemic conifers, respectively. In China, eight endemic conifer genera belong to the family Cupressaceae. Out of these genera, *Juniperus* is the largest one with 12 endemic species [9]. Most endemic *Juniperus* species are restricted to habitats with high elevations in southwest China, a global hotspot of conifer diversity and endemism [9,10].

To improve biodiversity conservation and management efforts, it is imperative to predict the impact of climate change on the geographic distribution of endangered species [11,12]. Species distribution models (SDMs) are used to predict the suitable habitat of endangered species. SDMs are considered useful tools for studying the impacts of climate change on biodiversity [13]. These models are also used to compare the current and projected future distribution of species based on current available environmental data and climatic change scenarios to define suitable habitats for implementing conservation actions to prevent species extinction [12,14]. Moreover, SDMs have been used to discover new populations of rare species and to propose conservation planning based on the available potentially suitable areas [15,16,17]. SDMs and migration-ability scenarios have been used to address the potential distributions and range shifts of conifer species, particularly the endemic *Abies* [18] and *Picea* [19]. 

*Juniperus* species play a remarkable role in sustaining several ecological services in the fragile Himalayan high land [20]. For example, *Juniperus* species help control soil erosion, to enhance soil fertility, and to purify air [20]. They are also used to improve the livelihoods of poor indigenous peoples inhabiting remote mountains and facing harsh climatic conditions [20]. Furthermore, local communities rely on Juniper species as sources of wood for construction, for shelter construction, for fuel, and for medicinal purposes, in addition to their value for ecotourism and recreation [20,21,22,23,24].

*Juniperus pingii* var. *pingii*, *J. tibetica,* and *J. komarovii*, are three endemic species restricted to southwest China (https://threatenedconifers.rbge.org.uk/) [25,26,27,28]. The first two species are categorized as vulnerable, while the third one is near threatened [28]. The region is also highly vulnerable to climate change impacts [26,27,28]. A decline in some *J*. *pingii* var. *pingii* habitats was inferred from observed intensive grazing [25]. *Juniperus tibetica* is also subjected to high threat levels due to slow growth in severe edaphic, climatic conditions and habitat destruction [29]. However, the population trend of *J*. *komarovii* is unknown, mainly because of the remoteness of the species localities [27]. Therefore, it is advised to conduct more surveys to evaluate the current geographical status and threats of the endemic *Juniperus* species [27].

For any conservation study under predicted climate change, it is recommended to incorporate other factors such as land-use and dispersal scenarios and to use IUCN guidelines [30]. To our best knowledge, there is no conservation assessment for the three *Juniperus* species under the climate change scenarios, and there is no previous rigorous conservation plan to define specific areas for their conservation. Therefore, there is an urgent need for a comprehensive study to aid field surveys to discover new or unknown populations and to address climate change impacts.

We hypothesize that the three endemic *Juniperus* study species are exposed to a risk of extinction in the future due to global warming. Therefore, our objectives were (1) to predict the potential distributions of three endemic *Juniperus* species (*Juniperus tibetica*, *J. pingii* var. *pingii*, and *J. komarovii*) under current and future climates along with dispersal scenarios, (2) to identify the critical environmental drivers that best explain their potential distributions, (3) to assess the extinction risk by estimating the percentage loss in the area of occupancy (AOO), and (4) to identify conservation priority areas for each species.

## 2. Materials and Methods

### 2.1. Species Occurrence Data and Conservation Status

We obtained 86 occurrence records for the three endemic *Juniperus* species from four sources: (1) Global Biodiversity Information Facility (GBIF, http://www.gbif.org/ Accessed April 2019), (2) Chinese Virtual Herbarium (CVH, http://www.cvh.ac.cn/), (3) National Specimen Information Infrastructure (http://www.nsii.org.cn/), and (4) field surveys carried out by the Biodiversity Research Group of the Chengdu Institute of Biology.

We removed unresolved names and synonyms based on the global conifer database [28]. We verified and filtered the occurrence points by removing duplicates, points outside southwest China, or outlier locations (e.g., lakes and land-use structures) using the global map of land cover in ArcGIS 10.3 (ESRI, Redlands, CA, USA).

The current conservation status of *Juniperus pingii* var. *pingii* and *J. tibetica* is considered vulnerable (VU) with declining populations, while *J. komarovii* is near threatened (NT) with an unknown population trend [28]. The current conservation assessments of the study *Juniperus* species were carried out between 2011 and 2013, and the population trends of the three target species are declining. Therefore, there is a need for rapid assessment of these species under climate change scenarios. 

### 2.2. Environmental Variables and Multicollinearity

A Digital elevation model (DEM) was downloaded from the U.S. geological survey (https://www.usgs.gov) at 30 arc-seconds spatial resolution. The 19 bioclimatic predictors of current and future climates were downloaded from the WorldClim v 2.1 (https://www.worldclim.org/data/worldclim21.html) at a 30-arcsecond resolution [31].

To evaluate the impact of projected climate change on the potential distribution of *Juniperus* species, we used two global general circulation models (GCMs): BCC-CSM1.1 (Beijing Climate Centre–Climate System Modelling 1.1, http://forecast.bcccsm.ncc-cma.net/web/channel-34.htm) and MIROC5 (Model for Interdisciplinary Research On Climate, http://www.icesfoundation.org/Pages/ScienceItemDetails.aspx?siid=181). BCC-CSM1.1 is widely used for Asian regions and performs well when describing vegetation dynamics compared to other GCMs [32]. Simultaneously, MIROC5 simulates extreme and summer precipitation better than other GCMs for the South Asian region [33]. We used an ensemble average of the two GCMs to reduce the uncertainty arising from a single GCM [34].

Two representative concentration pathways’ (RCP4.5 and RCP8.5) emission scenarios of 2070 (average of 2061–2080) were applied. The RCP4.5 pathway represents a moderate scenario, but RCP8.5 indicates a high scenario. The reason behind choosing these two RCP scenarios is because China is the largest emitting country of carbon dioxide [35]. 

We used the crop and mask functions of the “raster” package in R 3.5.3 [36] to clip the bioclimatic and elevation layers according to a China shapefile and then resampled the output into 60 arc-second (approximately 2 km) resolution, which is required for AOO calculation as described by [30]. Finally, based on the occurrence coordinates of each species, the values of bioclimatic and elevation variables were extracted for the analysis of multicollinearity.

To reduce overfitting of SDM models, we removed the highly correlated variables based on their variance inflation factor (VIF), which measures how strongly each predictor can be explained by the rest of the predictors [37]. To perform VIF analysis, we used the *vifcor* and *vifstep* functions of the package “usdm” [38] in R 3.5.3 to exclude the variables with VIF values more than five and a correlation threshold of 0.75, as recommended by [39]. The relative importance of predictor variables was estimated using the function *getVarImp* of the “SDM” package in R 3.5.3.

### 2.3. Models Construction and Ensemble Modelling

We used a recent ensemble-modeling (EM) technique by combining Generalized Linear Model (GLM), Random Forest (RF), Generalized Additive Model (GAM), and Boosted Regression Trees (BRT), which are characterized by high stability and transferability [40,41]. However, various approaches to model tuning and data processing contribute to performance heterogeneity, making it largely case-specific to the study purpose for the suitability of any given technique [42]. 

We projected each of the models under the current and future climate scenarios using 70% of the training data and 30% for evaluation [43]. The most effective SDMs require data on both species presence and available environmental conditions (pseudo-absence data); thus, for each species, the number of pseudo-absences was randomly sampled and equaled ten times the number of presences [44,45].

We used an ensemble modelling (EM) technique to reduce uncertainty in the model predictions. This is superior to standard models in optimizing the model performance and its transferability [39,42,43,46]. The ensemble models were weighted by the True Skill Statistic (TSS) using the “sdm” package in R 3.5.3 [37]. As a conservative approach and to minimize commission and omission errors, we used both maximum training sensitivity plus specificity (MTSS) and minimum training presence (MTP) as recommended thresholds [39,47]. The output maps produced from ensemble models were adjusted to the global land cover map, which was obtained at 1 km resolution for modeling of biodiversity [48], to filter the unsuitable or non-forest areas (inaccessible areas identified as suitable) to get a more precise prediction of suitable areas for the presence of *Juniperus* species. Later, we used threshold-independent metrics at 0.5 (i.e., 50% of the suitability score) because the predictions of both MTP and MTSS generated 100% loss in AOO under future scenarios. The threshold 50% generated maps with stable and gained areas, which could be assumed as a conservation planning tool for ex situ conservation. In other words, threshold-independent metrics (0.5) evaluate model performance using the “raw” probabilities of climate suitability for each grid cell in a prediction map: 0.01, 0.2, and so on, with higher numbers indicating greater suitability.

To evaluate the accuracy of models, we used the True skill statistic (TSS) and the area under the receiver-operating characteristic curve (AUC) [39].

### 2.4. AOO Estimation and Extinction Risk Assessment

A good advantage of the assessment approach employed in the current study is the use of the area of occupancy (AOO) as a strong predictor of extinction risks [30]. Furthermore, the use of the required spatial 2 km resolution from the beginning (i.e., before modelling analysis) to estimate AOO can help avoid potential errors induced by downscaling, geometric uncertainty, and grid orientation or origin [30,49,50]. We used the occurrence records and the extent of occurrence (EOO) computing function of “ConR” package in R 3.5.3 [51] to compute the α-hull EOO to create EOO shapefiles for each species. We used the alpha-hull method with the default buffer of 0.1 decimal degree (approximately 12 Km at the equator). This method has been suggested as an appropriate measure when the species has a disjunct distribution or when estimating range trends of the species [52]. It offers an overt way for eliminating cutoffs in a species range. Moreover, it helps to remove doubtful areas that are far off from species occurrences resulting from dispersal limitations. This method overcomes the limitations of the other methods as it eliminates bias due to sample size, and the spatial and temporal distribution of the occurrence records [53].

We used the EOO shapefiles in the “raster” package to crop and mask the suitable habitats of current and future output maps of each species separately. We adjusted to the global land cover map to filter the inaccessible areas identified as suitable. The resulting map was used for calculation of the AOO by counting the number of grid cells.

Since a dispersal scenario is an important aspect for in situ conservation [43], we applied two common dispersal scenarios (full dispersal and no dispersal). In the case of no dispersal, the predicted grid cells that occur in new areas in the future were considered unsuitable. For full dispersal, we assumed that there is no limitation to the dispersal capacity of a species, and grid cells were retained as part of the future distribution, even when they were not part of the potential present range [54]. 

We used the loss values in the projected AOO to assess extinction risks (proposed conservation status) according to the criterion A3(C) of the IUCN Red List, which indicates that a decline in the area of AOO is an indicator of a population reduction projected in the future [30]. Thus, we categorized species extinction risks as follows: least concern (LC = loss < 15%), near threatened (NT = loss > 15%), vulnerable (VU = loss > 30%), endangered (EN = loss > 50%), critically endangered (CR = loss > 80%), and extinct (EX = 100% loss) [30,54]. 

## 3. Results

### 3.1. Model Performance and Response to Climatic Changes

The ensemble models showed high accuracy and excellent performance; the averages of AUC and TSS were higher than 0.90 for *J. tibetica*, and *J. komarovii* and higher than 0.83 for *J*. *pingii* var. *pingii* (Table 1). The relative importance of predictor variables varied among *Juniperus* species. Temperature annual range (Bio7) was the most important factor predicting the potential distribution of *J*. *pingii* var. *pingii* (56.1% contribution).

**Figure 1:**
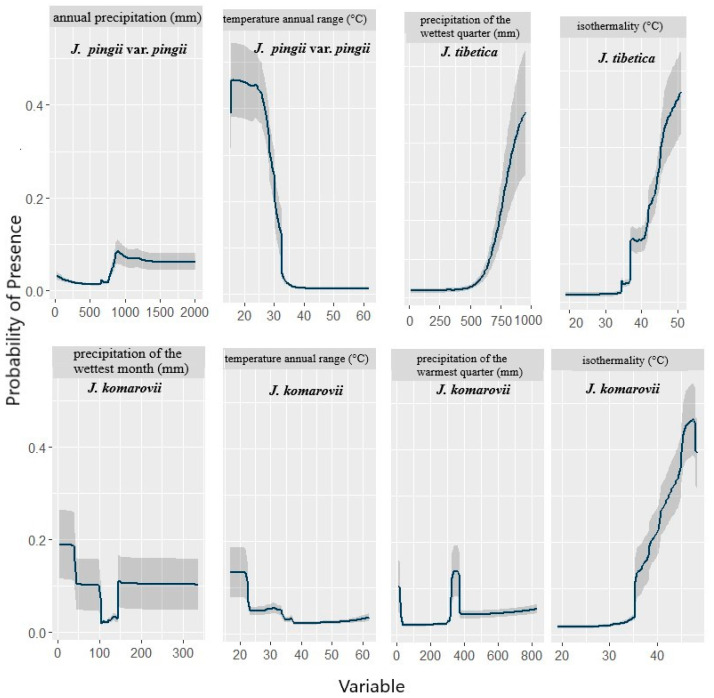
Response curves of the most important predictor variables used in distribution modelling of Juniperus species.

In comparison, isothermality (Bio3) was the most important predictor for the potential distribution of *J. tibetica* and *J. komarovii*, where it contributed 20.4% and 38.3%, respectively. (Table 1). In response to Bio7 (annual temperature range), the probability of the presence of *J. pingii* var. *pingii* showed sharp declines after 25 °C (Figure 1). Moreover, Bio7 showed a gradual decline in the cases of *J. tibetica* and *J. komarovii* and became almost constant after a temperature of 35 °C. Similarly, the likelihood of the presence of *J. tibetica* increased with the increase in precipitation of the wettest quarter (Bio16). The probability of the presence of *J. tibetica* and *J. komarovii* increased gradually with the increase in isothermality (Bio3) (Figure 1).

### 3.2. Potential Suitability and Projected AOO under Climate and Dispersal Scenarios

The potential changes in AOO were quite similar under the two emission scenarios (RCP4.5 and RCP8.5) and the two dispersal scenarios (full and no dispersal). Therefore, we illustrated only full dispersal and RCP4.5 scenarios (Table 2).

**Figure 2:**
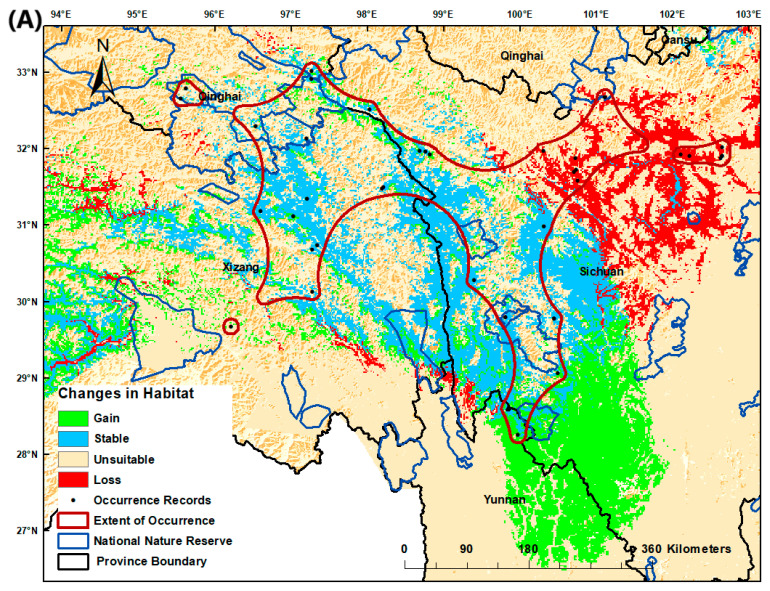

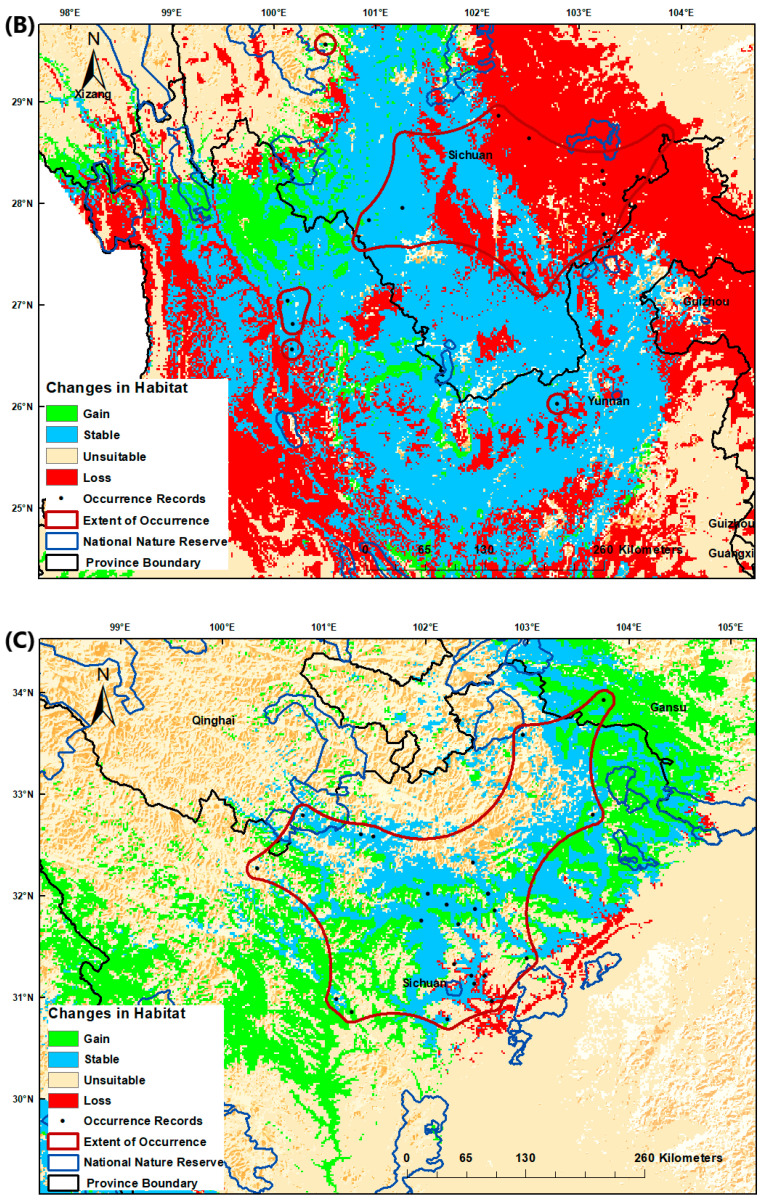
Potential habitat suitability under future RCP4.5 climate scenario for the (**A**) *Juniperus tibetica*, (**B**) *Juniperus pingii* var. *pingii*, and (**C**) *Juniperus komarovii* species based on threshold-independent metrics.

The three target *Juniperus* species showed 100% loss in their projected AOO under climatic and dispersal scenarios (Table 2). Although there are gained and stable habitats within the EOO in the threshold-independent analysis (Figure 2), the raw scores may be below the threshold for the AOO and therefore are not included in the future AOO prediction. The binary maps, based on a threshold-independent metric (0.5) that shows the changes in habitat revealed that most of the suitable habitats (gained and stable) for the three targeted species were mainly present outside their EOO, indicating range shift dynamics (Figure 2).

The results also showed that the best potential suitable habitats (gained and stable) for *J. tibetica* were predicted to be in the southeast parts of Sichuan Province (Figure 2A), while the predicted suitable habitats for *J. pingii* var. *pingii* (Figure 2B) and *J. komarovii* (Figure 2C) were mainly in the northern parts of Yunnan Province. These potential areas could be investigated for the possible occurrence of new or undiscovered populations of *Juniperus* species. Potential suitability maps of the target species under present and future climate scenarios are shown in Figure S1.

### 3.3. Potential Changes in Extinction Risk under Climate Scenarios

**Figure 3:**
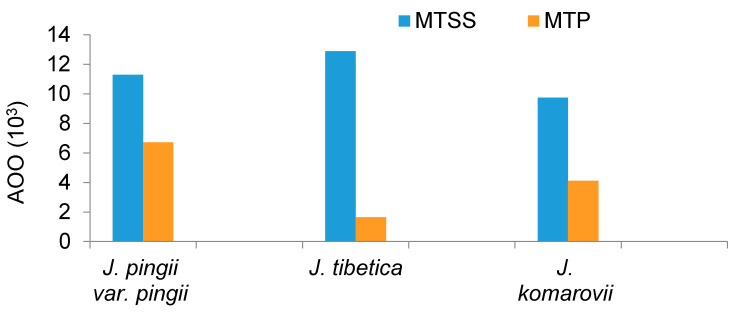
Potential present AOO of the three target *Juniperus* species derived from ensemble modelling based on two thresholds: maximum training sensitivity plus specificity (MTSS) and minimum training presence (MTP).

The projected status of all species was up-listed to the highest category of extinction risk “extinct”, based on 100% loss in the projected AOO (according to IUCN Red List Criterion A3 (C)) under both climate and dispersal scenarios (Table 2). The potential AOO showed high significant variation between the two thresholds (MTSS and MTP), particularly for *J. tibetica* (Figure 3).

The potentially suitable areas for each species were mostly located outside the national nature reserves (Figure 2). Thus, these areas should be considered priority conservation areas for in situ conservation of the study *Juniperus* species and further surveys.

## 4. Discussion

### 4.1. Distribution Modelling and Conservation Assessment

Climate is the main driver of plant species’ distribution [55,56,57]. Plant species inhabiting high elevations, such as Montane trees, are more vulnerable to extinction risks due to climate change [58,59,60,61]. Research has been conducted to study the potential role of climate change in the extinction risk of montane species [58,62,63,64,65,66]. However, more studies are needed to further understand the influence of climate change relative to other current threats [65], such as land-use change, using ensemble modelling techniques. Furthermore, the vulnerability and risk of habitat losses due to climate change for gymnosperm taxa in general and conifers in particular are not yet clear [67].

Studying the range shift, potential distribution, and migration ability in response to climate change was addressed in previous studies for some conifer species, particularly *Abies* and *Picea,* e.g., in [68,69]. However, the range shift or potential distribution of endemic *Juniperus* species in response to climate change is still not fully investigated. Therefore, the current study examines the impact of climate change on the range extent and extinction risk of endemic *Juniperus* montane tree species in the Tibetan Plateau and southwest China. The studied *Juniperus* species are known to be threatened by several environmental threats, such as land-use change, overexploitation, and invasive species [25,29]. For example, *J. tibetica* is the main high-altitude tree in large parts of the study area and the southeastern Tibetan Plateau, the species with the highest tree line in the world [70]. This species is considered an essential wood source to local communities who also utilize it as incense in Buddhist rituals [29,71]. Therefore, the species is under high pressure due to overexploitation.

The relative importance of the bioclimatic predictors varied among the investigated *Juniperus* species. However, the temperature-related variables, especially temperature annual range, were the most important variables controlling the distribution of the studied species, particularly *J. pingii* var. *pingii*. However, the probability of occurrence of *J. tibetica* showed a sharper decline after an increase in temperature compared to that of *J. pingii* var. *pingii* and *J. komarovii*. This indicates that *J*. *tibetica* could be more resilient to global warming than *J. pingii* var. *pingii*. Other studies have revealed a positive correlation between *J. tibetica* growth and winter season temperatures [72,73,74,75].

Isothermality, which indicates how the day-to-night temperatures fluctuate relative to the summer-to-winter fluctuations, contributed significantly as an important predictor for the distribution of both *J. komarovii* and *J. tibetica*. The probability of occurrence of *J. tibetica* and *J. komarovii* increased gradually with the increase of isothermality. The studied alpine species are sensitive to temperature variation [76]; therefore, isothermality is a critical determinant of their distribution. Isothermality has been reported as one of the most crucial factors correlated with the distribution of other alpine species, e.g., *Abies* spp., in the mountains of southwest China [77,78,79]. 

The three *Juniperus* species exhibited a narrow optimal range of temperature variation, which is also relatively lower than the predicted temperature increase in the area. This could explain the significant decline in the climatically suitable areas for the three species according to the future climatic scenarios. Therefore, future projections based on an increase in the annual temperature range could cause a substantial decline in the climatically suitable areas for the investigated species. 

Mountain species have a relatively low sensitivity to precipitation variability [77]. However, water availability-related variables, particularly precipitation of the wettest quarter (summer) and annual precipitation, contributed considerably as limiting determinants for the distribution of *J. tibetica* and *J. pingii* var. *pingii*, respectively. Also, the probability of the presence of *J. pingii* var. *pingii* will increase with the increase in annual precipitation. It has been reported that moisture availability through the pre-monsoon season positively influences the growth and recruitment of *Juniperus* species [80]. Other studies indicated that juniper species growth, particularly at higher elevations, has become adversely influenced by the increase in summer drought frequency during the recent few decades [10]. Moisture availability and wet conditions during the primary growing season (i.e., in summer) are advantageous for the growth of *Juniperus* species, which was confirmed by cambial phenology studies that revealed a stimulating effect of early growing season temperatures on *Juniperus* species cell formation [80]. The integration of physiological traits into ecological modelling would help explain the distribution pattern of species [81].

### 4.2. Impact of Climate Change on the Habitat Suitability and Projected AOO

Although species, in general, are likely to respond differently under climate change scenarios, the three studied *Juniperus* species were predicted to suffer a complete loss in their AOO under all combinations of climate and dispersal scenarios (4.5 and 8.5 RCPs, and full and no dispersal scenarios). Based on the MTSS threshold, only *J. komarovii* was projected to gain AOO by 2070. Projections of the current study revealed that the studied *Juniperus* species would be exposed to a continuous decline in their suitable habitat areas, particularly *J. pingii* var. *pingii* and *J. tibetica*, even under the moderate emission scenarios (i.e., RCP4.5). This suggests the urgent need for immediate actions such as ex situ conservation and afforestation of these species in stable or suitable areas, taking the results of the current study as a guideline tool.

Species dispersal is greatly controlled by both suitable habitat availability and connectivity [82]. It is crucial to consider the differences in species dispersal abilities and responses to climate change for understanding how the potential habitat configuration, which is the spatial arrangement of habitats at a given time, may hinder or foster climate-driven distributions and range shifts [83].

The shift towards the southeast of the current EOO is projected for *J. tibetica* and *J. pingii* var. *pingii*, while a southwest shift is projected for *J. komarovii*. Conservation planning considers the shift in distribution of suitable habitats, which will mostly be outside the boundaries of the existing EOO. Therefore, re-zonation and design of the protected areas to accommodate changes in species habitat patches is highly recommended. Also, the establishment of habitat corridors may be required to secure proper habitat connectivity. 

### 4.3. Risk of Extinction under Climate and Dispersal Scenarios

The current IUCN conservation status for both *J. pingii* var. *pingii* and *J. tibetica* has been declared vulnerable (VU) [25,29], and that for *J. komarovii* is near threatened (NT) [27]. The projection of our study revealed a trend of decline in the suitable habitat and complete losses of the current AOOs of the three species by 2070. Therefore, the status of these *Juniperus* species will be up-listed to the highest category of the extinction risk, which is “extinct”, under all combinations of climate and dispersal scenarios. Although the outcomes revealed a future loss of AOO by 2070, caution is needed regarding the claim of future complete loss of habitats. There might be possibilities of adaptation to climate change through genetic adaptation or phenotypic plasticity of the *Juniperus* species. Besides, the species might develop long-distance migration to areas with high habitat-suitability. 

This study did not account for future environmental and climate changes on habitat configuration and connectivity. Changes in habitat configuration can reduce connectivity amongst areas inhabited by the various populations of a particular species [84], which could lead to a reduction in the species capability to endure environmental threats and might accelerate its extinction risk [85]. Therefore, retaining the available suitable habitat for species and enhancing habitat connectivity are the foremost conservation targets [86,87]. Consequently, it is recommended to use the landscape metrics (e.g., number and size of patches, patch configuration, and connectivity) for future assessment of climate-change impacts on the capability of dispersal and persistence of the studied *Juniperus* species. 

The potential AOOs of *J. tibetica* and *J. komarovii* showed variation under the two different thresholds (MTSS and MTP). An investigation of the outcomes from using different thresholds is important, especially when dealing with assessing potential range shift of species [88]. We have used MTP and MTSS threshold rules to provide liberal (the former) and conservative (the latter) predictions of climatic suitability for the investigated species. For conservation planning, it is important to account for the best- and the worst-case scenarios, which can be recognized using these two threshold rules. Accounting for both threshold rules provides a balanced approach for SDM applications in conservation biology when the goal is to assess potential climatic suitability in new geographies or the future [89].

The use of future projections under climate change scenarios in conservation planning needs to be considered on a species-by-species case [88,90]. The high-potential suitable habitat for *J. tibetica* was predicted to be in the eastern-central parts of Sichuan Province, while the predicted suitable habitats for *J. pingii* var. *pingii* and *J. komarovii* were mainly in the northern parts of Yunnan Province. While some existing national nature reserves cover the projected range of distribution of the studied *Juniperus* species, new protected areas should be established to cover the projected changes in distribution, particularly for the conservation of *J. komarovii*. The establishment of nature reserves that include areas that have been predicted as suitable for more than one of the investigated *Juniperus* species is highly recommended. Care should be taken when establishing such conservation sites, as management objectives set for a particular species may not result in advantages to other species’ perseverance or can even threaten the existence of other species [91,92].

### 4.4. Conservation Implications

Conservation efforts should prioritize these three species in any future creation of protected areas and any other relevant conservation actions, particularly for *Juniperus pingii* var. *pingii*, which is not well represented in the current protected areas. Any future conservation actions should consider the southeast of Sichuan Province and the northern-central part of Yunnan as priority areas for in situ conservation of the threatened *Juniperus* species. This is especially important as the current study revealed that these areas could include locations suitable for more than one of the investigated *Juniperus*. Ex situ conservation activities directed towards increasing representation of the three Juniperus species in China‘s six major Botanic gardens are highly recommended. Also, it is advisable that these species should be prioritized in China’s National Plan for the Protection and Utilization of Biological Species Resources plans for species-protection projects. Areas of projected occurrence of the studied *Juniperus* species identified through the SDM outcomes of the current study can be used to facilitate surveys and investigations aimed at detecting new populations of *Juniperus* species. Previous studies have recommended conducting an intensive survey to detect new populations of the studied *Juniperus* species, e.g., [27]. Outcomes from SDM predictions of suitable habitats have been used to uncover new populations of rare or endangered species [15,16,17,93]. 

The projected loss in these three *Juniperus* habitats will impact the provision of ecosystem services in the region. Among the ecosystem services that could be affected are the shelter provided for many other rare and threatened animal species, carbon sequestration enhancement, and facilitation of other species [71]. A more in-depth analysis that evaluates the projected consequences and impacts of losses in the investigated *Juniperus* species on other associated species and the sustainability of ecosystem functions and services in the region is especially required. 

It should be noted that the establishment of nature reserves and protected areas will not guarantee the preservation and sustainability of the investigated *Juniperus* species populations. *Juniperus* species are a component of vulnerable ecosystems due to many environmental changes triggered by human activities. For example, *J. pingii* var. *pingii* populations are declining due to overgrazing [25]. The inaccessibility of areas where the *J. komarovii* population has been recorded makes it difficult to survey and to fully assess the threats that these populations currently face [27]. Evaluating the threats to nature reserves from human activities in the surrounding areas is crucial for ensuring the effectiveness of these reserves in achieving their conservation management objectives [29]. 

The projected changes in AOOs of the studied *Juniperus* species were corrected for the current transformation of the land. However, the projections did not account for the potential changes in land use/land cover and how that would influence the AOOs. Also, the impact that this will exert on habitat configuration and connectivity was not considered. Hence, the impact on the dispersal capability of species was not addressed. Therefore, conservation policies and planning directed at these species need to consider the shared pattern of climate change and land use to implement conservation actions successfully.

## 5. Conclusions

The projections in the current study revealed that the studied *Juniperus* species would be exposed to continuous decline in their suitable habitat areas and increase in extinction risk under the different combinations of climate change and dispersal scenarios. The projected loss percentage in the AOOs under all combinations of climate and dispersal scenarios would result in up-listing in the status of *Juniperus* species to a higher extinction risk category. The implications of these findings are significant for the adoption of appropriate long-term measures for *Juniperus* species conservation.

Future conservation efforts should give higher priority to the creation of protected areas for the conservation of *J. pingii* var. *pingii* and *J. komarovii*, especially because these species are currently not well represented in protected areas. The southeast of Sichuan Province and the northern-central part of Yunnan are recommended as priority conservation areas for in situ conservation action of the threatened *Juniperus* species.

The outcomes from the SDM predictions can be valuable in supporting resolutions for on-ground conservation challenges. Field surveys should be conducted in areas that are projected to provide climatically suitable habitats for the *Juniperus* species. Such surveys could detect new populations of species, particularly *J. komarovii,* which has been reported to receive less attention in surveying its populations due to the difficulty to access its habitats [71]. 

Furthermore, we recommend conservationists to consult various threshold determination rules in any future conservation studies of different taxa. The use of MTP and MTSS threshold rules provides liberal and conservative predictions of climatic suitability for the investigated species. Thus, the thresholds account for the best and worst-case scenarios in the predicted suitable areas and hence in the potential status of extinction risk of the specific taxon. This helps to provide a balanced approach for SDM applications in conservation biology, where the goal is to assess potential climatic suitability in new geographies or in the future [89].

Authorizing conservation efforts or management policies concerning the studied species should combine a pattern of climate and land-use changes. Therefore, the inclusion of future land-use change scenarios should be recommended in future integrated modelling approaches aimed at assessing the impacts of future climate and environmental changes on species distribution and availability of suitable habitats.

## Figures and Tables

**Table 1:** The importance of predictor variables in the potential distribution of *Juniperus* species: correlated variables with variance inflation factor (VIF) values > 5 and a correlation threshold of 0.75 were removed to avoid multicollinearity problems.

Species	Variable Importance	Bioclimatic Variables	Ensemble-Model Accuracy		Ensemble-Model Threshold			
		VIF	Min. Value	Max. Value	AUC	TSS	MTSS	MTP
*Juniperus pingii* var. *pingii*	Bio7 (56.1%)	2.09	21.75	33.65	0.92	0.83	0.27	0.71
	Bio12 (17.5)	2.97	573.5	987				
*Juniperus tibetica*	Bio3 (20.4%)	1.32	34.44	45.62	0.96	0.91	0.14	0.73
			
	Bio16 (16.8%)	1.3	295	419.2				
*Juniperus komarovii*	Bio3 (38.3%)	1.59	30.8	46.65	0.97	0.93	0.36	0.53
	Bio13 (20%)	4.53	108.8	147				
	Bio18 (19.8%)	4.07	298.8	380.8				
	Bio7(16%)	2.08	27.62	36.9				

Bio7 = temperature annual range (°C); Bio12 = annual precipitation (mm); Bio13 = precipitation of the wettest month (mm); Bio16 = precipitation of the wettest quarter (mm); Bio3 = isothermality (°C); Bio18 = precipitation of the warmest quarter (mm); AUC = area under the curve; TSS = true-skill statistics; MTSS = maximum training sensitivity plus specificity; and MTP = minimum training presence.

**Table 2:** Current and proposed extinction risk (conservation status) and area of occupancy (AOO) changes under current and future (2070) potential distributions for the target *Juniperus* species in the representative concentration pathway (RCP) 4.5 and RCP8.5 scenarios and assuming either limited dispersal or full dispersal scenarios: changes in the projected AOO were based on MTSS and MTP.

Species	Number of Records	Current IUCN Status	Future Climate (2070)					
			Population Trend	MTSS Threshold		MTP Threshold		Potential Status Change
				AOO Change (%)	Potential Status *	AOO Change (%)	Potential Status	
*Juniperus pingii* var. *pingii*	20	VU	Declining	100% Loss	EX	100% loss	EX	Up-listed
*Juniperus tibetica*	41	VU	Declining	100% Loss	EX	100% loss	EX	Up-listed
*Juniperus komarovii*	25	NT	Unknown	26% Gain	LC	100% loss	EX	Up-listed

* Proposed status based on the IUCN Red List Criterion A3(C), which indicates that a decline in area of AOO or habitat quality means a projected population reduction in the future (up to a maximum of 100 years) [22]. EX: Extinct, VU: Vulnerable, NT: Near Threatened, and LC: Least Concern.

## Data Availability

Data are contained within the article or supplementary material.

## Supplementary Materials

Supplementary material is available online at https://www.mdpi.com/2079-7737/10/1/63/s1. Figure S1: Potential habitat suitability maps of the three target Juniperus species under present and future (RCP 4.5) climatic scenarios. The green color indicate to suitable habitat. EOO is the extent of occurrence (EOO) of species.
