# Supplementary material for: Endemic Juniperus Montane Species Facing Extinction Risk under Climate Change in Southwest China: Integrative Approach for Conservation Assessment and Prioritization
Source: Biology (Basel). 2021 Jan 17;10(1):63. doi: 10.3390/biology10010063 (PMC7830502; doi:10.3390/biology10010063)
Supplement: Supplementary file 1 [file biology-10-00063-s001.pdf]

biology

Supplementary Materials

Endemic *Juniperus* Montane Species Facing Extinction Risk under Climate Change in Southwest China: Integrative Approach for Conservation Assessment and Prioritization

Mohammed A. Dakhil, Marwa Waseem A. Halmy, Walaa A. Hassan, Ali El-Keblawy, Kaiwen Pan and Mohamed Abdelaal

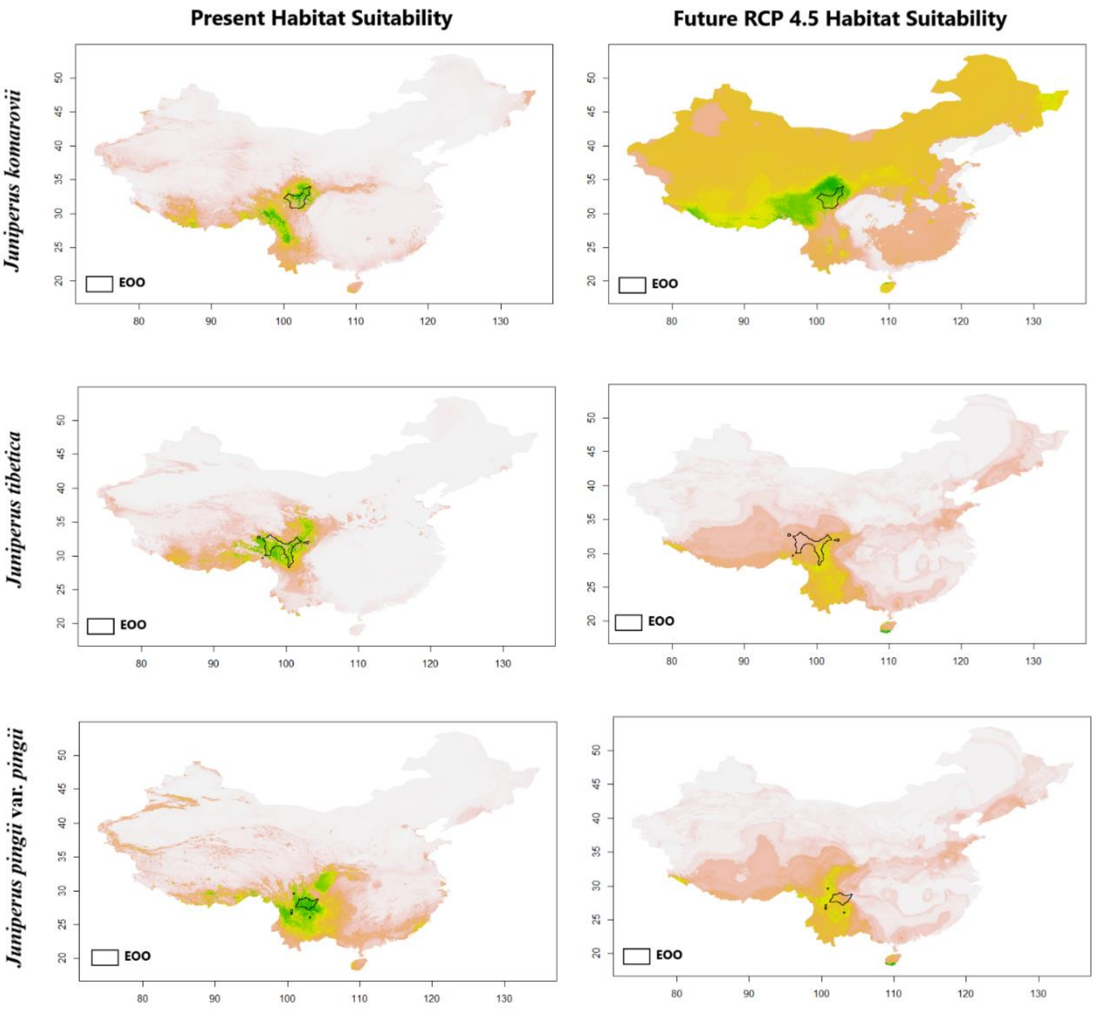

Figure S1. Potential habitat suitability maps of the three target *Juniperus* species under present and future (RCP 4.5) climatic scenarios. The green color indicate to suitable habitat. EOO is the extent of occurrence (EOO) of species.
